# Arthritic Microenvironment‐Dictated Fate Decisions for Stem Cells in Cartilage Repair

**DOI:** 10.1002/advs.202207715

**Published:** 2023-07-30

**Authors:** Songlin He, Haotian Deng, Peiqi Li, Jingjing Hu, Yongkang Yang, Ziheng Xu, Shuyun Liu, Weimin Guo, Quanyi Guo

**Affiliations:** ^1^ School of Medicine Nankai University Tianjin 300071 China; ^2^ Institute of Orthopedics the First Medical Center Chinese PLA General Hospital Beijing Key Lab of Regenerative Medicine in Orthopedics Key Laboratory of Musculoskeletal Trauma & War Injuries PLA Beijing 100853 China; ^3^ Department of Gastroenterology Institute of Geriatrics Chinese PLA General Hospital Beijing 100853 China; ^4^ Department of Orthopaedic Surgery Guangdong Provincial Key Laboratory of Orthopedics and Traumatology First Affiliated Hospital Sun Yat‐Sen University Guangzhou Guangdong 510080 China

**Keywords:** articular inflammatory microenvironment, cartilage repair, fate decision, spatial and temporal regulation, stem cells

## Abstract

The microenvironment and stem cell fate guidance of post‐traumatic articular cartilage regeneration is primarily the focus of cartilage tissue engineering. In articular cartilage, stem cells are characterized by overlapping lineages and uneven effectiveness. Within the first 12 weeks after trauma, the articular inflammatory microenvironment (AIME) plays a decisive role in determining the fate of stem cells and cartilage. The development of fibrocartilage and osteophyte hyperplasia is an adverse outcome of chronic inflammation, which results from an imbalance in the AIME during the cartilage tissue repair process. In this review, the sources for the different types of stem cells and their fate are summarized. The main pathophysiological events that occur within the AIME as well as their protagonists are also discussed. Additionally, regulatory strategies that may guide the fate of stem cells within the AIME are proposed. Finally, strategies that provide insight into AIME pathophysiology are discussed and the design of new materials that match the post‐traumatic progress of AIME pathophysiology in a spatial and temporal manner is guided. Thus, by regulating an appropriately modified inflammatory microenvironment, efficient stem cell‐mediated tissue repair may be achieved.

## Graphical abstract

Stem cells recruited at the defect site are characterized by overlapping lineages and uneven effectiveness. The inflammatory microenvironment plays a decisive role in guiding the fate of stem cells. Design strategies along with the evolution of inflammatory microenvironment are summarized and effective drug strategies are highlighted. Moreover, a design trend for new generation of tissue engineering repair materials is proposed.

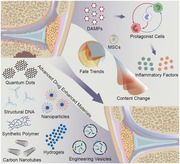

## 1 Introduction

Articular cartilage injury is an important problem worldwide.^[^ ^1^ ^]^ Articular cartilage is a lymphatic connective tissue that does not contain blood vessels or nerves. It is particularly difficult for articular cartilage within load‐bearing joints to recover normal function following injury,^[^ ^2^ ^]^ as disturbances of the articular inflammatory microenvironment (AIME) result in incomplete cartilage repair.^[^ ^3^ ^]^ In fact, AIME reconstruction determines the repair outcome within 3 months after injury,^[^ ^4^ ^]^ while the evolution of the AIME is associated with a systemic inflammatory response and a cytokine storm.^[^ ^5^ ^]^

Currently, only surgery and tissue engineering regeneration can repair damaged cartilage.^[^ ^6^ ^]^ The strategy of creating cartilage through tissue engineering using cells and exogenous stimuli has progressed with respect to articular cartilage repair. This is one of the most promising methods for treating articular cartilage injury.^[^ ^7^, ^8^ ^]^ Moreover, stem cell‐based tissue engineering offers a new therapeutic approach for patients.^[^ ^9^ ^]^

Two types of stem cells are used in tissue engineering: endogenous stem cells recruited from in situ tissue and exogenous stem cells that rely on tissue digestion and adherence.^[^ ^10^ ^]^ However, the mesenchymal stem cells (MSCs) used for these two methods are heterogenous, and the overall effectiveness of stem cells and the problem of overlapping lineages contribute to the uncertainty of tissue repair.^[^ ^11^ ^]^ In this review, we discuss the types of stem cells that contribute to articular cartilage repair and provide an overview of their fate. Subsequently, we describe the changes that occur in the microenvironment of the injured cartilage as inflammation develops and the effects of these changes on stem cell regeneration. We believe that the AIME plays a decisive role in the fate of stem cells and identify the key factors that determine the fate of stem cells at each time point during cartilage repair. The goal is for endogenous or exogenous stem cells to differentiate into cartilage by regulating the AIME, maximizing the potential of their fates, and achieve complete repair. Finally, we discuss the opportunities and challenges arising from the use of AIME deconstruction technologies, which will enable us to design a new generation of tissue engineering materials.

## 2 Stem Cell Positions and Fate Trends

**Figure 1:**
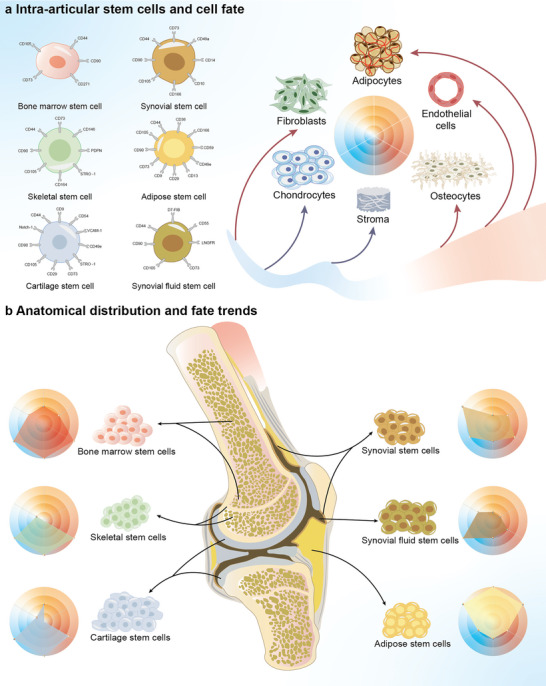
Anatomical location and fate of stem cells. a) Surface markers and the fate of stem cells. Stem cells express CD105, CD90, CD73, and CD44 on their surface but lack CD45, CD31, CD34, CD14, CD11b, CD79a, CD19, and HLA‐DR. Stem cells in the joint cavity experience six different outcomes. Adverse outcomes include fibroblasts, adipocytes, vascular endothelial cells, and osteocytes. The ideal outcome is differentiation into cartilage and cartilage matrix. b) Anatomical location and fate of stem cells in the articular cavity. The vertices in the radar map correspond to the fate of the stem cells in the AIME. The points on each branch of the radar graph represent the trend of the stem cells toward this fate. BMSCs are primarily distributed in the joint cavity and the growth plate, with strong osteogenic, chondrogenic, and matrix formation abilities. SSCs can only differentiate into chondrocytes, osteocytes, and stromal cells. The differentiation of SFSCs is similar to that of SMSCs, but their chondrogenic ability is greater. CSPCs have no tendency for angiogenic differentiation and are characterized by strong osteogenic, chondrogenic, and stromal abilities. ADSCs have a low chondrogenic differentiation ability but can readily differentiate into fibrocytes, adipocytes, or vascular endothelial cells.

Based on cell surface antigens, labeled MSCs are defined as plasticity‐adherent cells that express CD105, CD90, CD73, and CD44, while lacking CD45, CD31, endothelial or primitive hematopoietic (CD34) markers, monocyte (CD14 or CD11b), B cell (CD79a or CD19) markers, and human leukocyte antigen (HLA) class II (HLA‐DR) surface antigens.^[^ ^12^, ^13^ ^]^ Stem cells from different parts of the body express different surface markers (**Figure** **1**a). There are five stem cell types in situ in the articular cavity, which include bone marrow mesenchymal stem cells (BMSCs), adipose‐derived mesenchymal stem cells (ADSCs), synovial mesenchymal stem cells (SMSCs), synovial fluid mesenchymal stem cells (SFSCs), cartilage stem/progenitor cells (CSPCs), and skeletal stem cells (SSCs). Herein, we summarize the specific surface markers of stem cells from different anatomical locations as well as the fate of different stem cells during the process of cartilage repair (Figure 1b). The locations and characteristics of dormant stem cells are listed in **Table** **1**.**^[^** ^14^, ^15^, ^16^, ^17^, ^18^, ^19^, ^20^, ^21^, ^22^, ^23^, ^24^ ^]^

**Table 1:** Dormant stem cell locations and characteristics

Cell type	Specific surface markers	Dormant locations	Cell fates	Characteristics	References
BMSCs^a)^	CD45^−^, CD271^+^, CD140a^low/−^, CD90^+^, CD105^+^, CD271	Subchondral bone Bone marrow	Cartilage; Bone; Fat	Strong osteogenic and chondrogenic abilities	[14]
SMSCs^a)^	CD105^+^ and CD166^+^ CD10^+/low^, CD14^+^, CD44^+^, CD49a^+^, CD73^+^	Synovium	Cartilage; Bone; Fat	Early response of cartilage injury Chondrogenic ability stronger than that of BMSCs Fibroblast differentiation tendency	[15]
SFSCs^a)^	D7‐FIB^+^, CD13^+^, CD105^+^, CD55^+^, LNGFR^+^,^a)^ CD45^−^, BMPRIA^−^,^a)^ CD10^low/−^	Synovial fluid	Cartilage; Bone; Fat	Clonality higher than that of SMSCs	[16]
ADSCs^a)^	CD9^+^, CD10^+^, CD13^+^, CD29^+^, CD44^+^, CD49e^+^, CD59^+^, CD105^+^, CD166^+^, CD36^+^	IFP^a^	Cartilage; Bone; Fat	Low cost and easy access Adipose tissue differentiation tendency	[13, 17]
CSPCs^a)^	CD9^+^, CD29^+^, CD166^+^, CD49e^+^, CD54^+^, Notch‐1^+^, STRO‐1^+^,^a)^ VCAM‐1^+a)^	Cartilage	Cartilage; Bone; Fat	Chondrogenic ability higher than that of ADSCs	[18, 19, 20, 21]
SSCs^a)^	PDPN^+^, CD73^+^, CD164^+^ STRO‐1^+^, CD146^+^	Growth plate subchondral bone	Cartilage; Bone;	Differentiate only into cartilage and bone cells	[22, 23, 24]

^a)^ ADSCs, adipose‐derived mesenchymal stem cells; BMPRIA, bone morphogenetic protein receptor type IA; BMSCs, bone marrow mesenchymal stem cells; CD, cluster of differentiation; CSPCs, cartilage stem/progenitor cells; IFP, infrapatellar fat pad; LNGFR, low‐affinity nerve growth factor receptor; PDPN, podoplanin; SFSCs, synovial fluid mesenchymal stem cells; SMSCs, synovial mesenchymal stem cells; SSCs, skeletal stem cells; VCAM‐1, vascular cell adhesion protein 1; STRO: Stromal cell antigen.

### 2.1 Bone Marrow Mesenchymal Stem Cells

BMSCs originate in the bone marrow cavity. Their stemness has been demonstrated by the formation of ectopic bone tissue from a single cultured BMSC.^[^ ^25^ ^]^ Ghazanfari et al. demonstrated that BMSCs expressing CD45^−^, CD271^+^, CD140a^low/−^, CD90^+^, and CD105^+^ have the potential for trilineage differentiation into adipose, bone, and cartilage tissue.^[^ ^14^ ^]^ CD271 is the most specific surface molecule used for the isolation of BMSCs.^[^ ^26^ ^]^ Compared with umbilical cord MSCs and ADSCs, BMSCs exhibit lower methylation chondrogenic production induced by the microenvironment and highly express COL2A, COL10A, ACAN, PTHR1, and DLX6, with a stronger chondrogenic ability. This indicates that the microenvironment surrounding BMSCs is closely associated with the expression of a specific endochondral differentiation pattern in the BMSCs.^[^ ^27^, ^28^ ^]^ BMSCs are located in the subchondral bone, in marrow, and on the inner surface of the bone. They directly participate in the process of bone and cartilage remodeling without having to migrate to the periphery of vascular tissue.^[^ ^29^ ^]^ Adult chondrogenesis‐inducing injuries that can trigger the pathway of endochondral ossification cause BMSCs to migrate to the site of the injury to form a unique niche in the microenvironment, which in turn differentiate into an early cartilaginous tissue.^[^ ^28^, ^30^, ^31^ ^]^

### 2.2 Adipose‐Derived Mesenchymal Stem Cells

ADSCs can differentiate into a wide range of cell lineages, such as bone, cartilage, vascular endothelium, liver, and nerve cells.^[^ ^32^ ^]^ Within the joint cavity, ADSCs originate from within the infrapatellar fat pad (IFP), where most stem cells are latent in the stromal vascular fraction (SVF) and they have some clinical application.^[^ ^33^ ^]^ Both ADSCs and BMSCs are multipotent; however, BMSCs are more committed to osteogenic and chondrogenic lineages, whereas ADSCs are more committed to the adipogenic lineage. ADSCs tend to differentiate into adipocytes and vascular endothelial cells and are affected by the SVF microenvironment.^[^ ^34^, ^35^ ^]^ When exposed in vitro to transforming growth factor beta (TGF‐β), ascorbic acid, and dexamethasone, ADSCs expressing CD9^+^, CD10^+^, CD13^+^, CD29^+^, CD44^+^, CD49e^+^, CD59^+^, CD105^+^, CD36^+^, and CD166^+^ are more inclined to differentiate into chondrocytes.^[^ ^17^ ^]^ CD36^+^ and CD106^−^ are specific markers for ADSCs.^[^ ^13^ ^]^ Compared with BMSCs, ADSCs are easier to obtain and exhibit higher proliferation ability.^[^ ^36^ ^]^ However, the application of ADSCs to tissue engineering depends more on the regulation of the microenvironment, which induces ADSCs to undergo programmed chondrogenic differentiation. For example, various factors, such as bone morphogenetic protein (BMP)−6, TGF‐β3, insulin‐like growth factor 1 (IGF‐1), and the presence of an alkaline environment substantially affect the collagen type I alpha 1 (COL1A1) and alpha 2 (COL2A1) contents of the cartilage tissue formed by ADSCs.^[^ ^37^ ^]^

### 2.3 Synovial Mesenchymal Stem Cells

During the cartilage tissue repair process, SMSCs enter the superficial area to assist the resident cartilage progenitor cells to repair the cartilage defects.^[^ ^38^ ^]^ When cartilage is injured, more SMSCs are released from synovial tissue compared with adipose tissue, as SMSCs more readily enter the cartilage tissue.^[^ ^39^ ^]^ Of note, immediately separated synovial cells express CD10, CD13, CD14, CD34, CD44, CD45, CD49a, CD62e, CD73, and HLA‐DR surface markers. After passage, CD14, CD34, CD45, CD62e, and HLA‐DR disappear, CD105 and CD166 appear, and CD10, CD14, CD44, CD49a, and CD73 exhibit increased expression.^[^ ^15^ ^]^ SMSCs show little change from passage 1 to 8 and are characterized by the potential for chondrogenesis and differentiation.^[^ ^15^ ^]^ This shows the difference between resting and active SMSCs. When the microenvironment changes, SMSCs re‐adopt the characteristics of cartilage stem cells. In the specific matrix microenvironment of an extracellular matrix (ECM) occupied by SMSCs, adult SMSCs are restored to optimal vitality, thereby promoting cartilage regeneration.^[^ ^40^ ^]^

### 2.4 Synovial Fluid Mesenchymal Stem Cells

During the repair process, SFSCs, like SMSCs, are mainly responsible for repairing the superficial layer of articular cartilage.^[^ ^38^ ^]^ SFSCs originate from an adjacent synovium or from a superficial layer of the cartilage.^[^ ^41^ ^]^ Whether in normal synovial fluid or in the synovial fluid of osteoarthritis (OA) patients, SFSCs express the following markers: D7‐FIB^+^, CD13^+^, CD105^+^, CD55^+^, low‐affinity nerve growth factor receptor (LNGFR)^+^, CD45^−^, BMP receptor type IA (BMPRIA)^−^, and CD10^low/−^.^[^ ^16^ ^]^ In the normal human knee joint, SFSCs account for 0.025% of the total cells in the joint.^[^ ^42^ ^]^ Compared with BMSCs, SFSCs have a tendency to form cartilage that may be related to higher hyaluronic acid (HA) content in the microenvironment around SFSCs.^[^ ^43^, ^44^ ^]^ Because of the lack of a guiding microenvironment in the joint synovial fluid, stem cell fate cannot be guided. Compared with SMSCs, SFSCs are characterized by higher clonality and a more uncertain differentiation orientation.^[^ ^45^, ^46^ ^]^

### 2.5 Cartilage Stem/Progenitor Cells

Although articular cartilage lacks inherent repair ability, it has been proved that the superficial layer of articular cartilage contains CSPCs.^[^ ^47^, ^48^ ^]^ In the resting state, cartilage stem cells, such as CSPCs, participate in the maintenance of homeostasis.^[^ ^49^ ^]^ CSPCs express many specific markers, including CD9, CD29, CD166, CD49e, and CD5. In the OA microenvironment, CSPCs also express Notch‐1, STRO‐1, and vascular cell adhesion protein 1 (VCAM‐1). CSPCs isolated based on these markers have the potential to differentiate into cartilage, fat, and bone, whereas their chondrogenic ability is higher than that of ADSCs.^[^ ^18^, ^19^, ^20^ ^]^ Downregulation of the Runt‐related transcription factor 2 gene enhances the chondrogenic differentiation potential of CSPCs.^[^ ^21^ ^]^ Following mechanical injury of the cartilage or in an OA environment, CSPCs are released from dormancy, participate in the early stage of cartilage repair, and upregulate the expression of ECM‐related proteins.^[^ ^50^, ^51^, ^52^ ^]^ Additionally, the 3D microenvironment, ECM components, TGF‐β3, and BMP‐6 can significantly influence the fate of CSPCs.^[^ ^20^ ^]^ In fact, the chondroprotective ability of CSPCs may be enhanced by inhibiting matrix degradation.^[^ ^21^ ^]^

### 2.6 Skeletal Stem Cells

In recent years, researchers have further examined bone progenitor cells. For instance, Chan et al. identified SSCs.^[^ ^53^ ^]^ SSCs express podoplanin (PDPN), CD146, CD73, and CD164 on their surface and are derived from fetal bone, adult bone, BMP‐2‐treated human adipose stroma, and skeletally differentiated induced pluripotent stem cells.^[^ ^22^ ^]^ Although SSCs may be enriched with the cell surface markers STRO‐1 and CD146^+^, individual cell‐specific markers that are expressed by SSCs remain undefined.^[^ ^23^, ^24^ ^]^ SSCs can only differentiate into progenitor cells in the cartilage and matrix and do not produce adipocytes or endothelial cells. SSCs can also form bone and bone marrow matrix in nonskeletal tissues. The fate of SSCs may be guided by the microenvironment irrespective of whether it involves a change from bone to cartilage or vice versa.^[^ ^53^ ^]^

## 3 Determinants of Stem Cell Fate in the AIME

**Figure 2:**
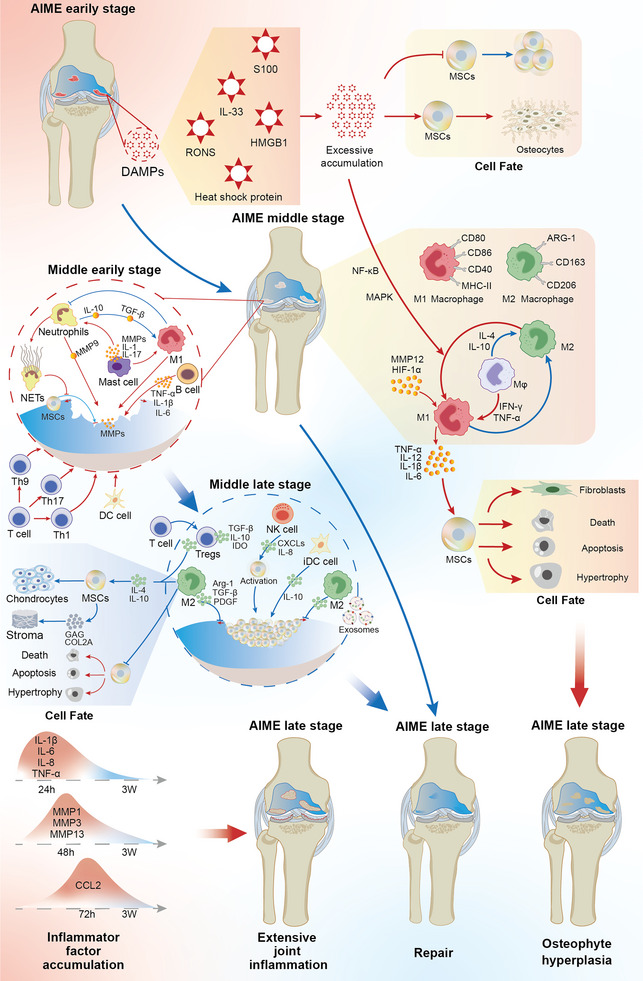
AIME evolution and crosstalk with stem cell fate. Early AIME involves the release of danger signals (DAMPs). An excess accumulation of danger signals leads to the differentiation of stem cells into osteocytes and can affect the polarization of macrophages at the intermediate stage of the AIME. The early protagonist in the middle stage of the AIME is the M1 macrophage. Neutrophils secrete NETs, whereas mast cells, B‐cells, T‐cells, and mature DCs secrete cytokines that inhibit the attachment of stem cells and clear the necrotic tissue from the site of the joint injury. The excessive polarization of M1 macrophages results in poor outcomes (such as the differentiation of stem cells into fibrocytes, death, apoptosis, hypertrophy, and ultimately, an imbalance in the AIME), accompanied by the generation of osteophytes and fibrous tissue. In the late phase of the middle AIME stage, the M2 macrophages become the protagonists. NK and Treg cells assist the M2 macrophages in recruiting and activating stem cells. M2 macrophages also promote the differentiation of stem cells into cartilaginous and stromal tissues while suppressing the adverse outcomes of stem cells. The late stage of the AIME is dominated by the clearance of inflammatory factors, such as IL‐1β, IL‐6, IL‐8, and TNF‐α, within the first 24 h following injury. The MMPs then peak at 24 h after the inflammatory factors have been identified, whereas CCL2 peaks within 72 h. These cytokines approach normal levels by ≈3 weeks. If the accumulated inflammatory factors are not cleared, they can lead to the development of generalized joint inflammation.

Because the joint cavity is a closed environment, inflammation triggered by most cartilage injuries is a form of aseptic inflammation.^[^ ^54^ ^]^ The AIME primarily includes damaged cells, vasculature, infiltrating immune cells, danger (inflammatory) signals, and proinflammatory molecules (such as cytokines, chemokines, and reactive oxygen species (ROS)). The AIME plays three main roles: release of danger signals, recruitment and polarization of inflammatory cells, and removal of inflammatory factors. Early inflammation promotes the clearance of necrotic tissue, activation of immune cells, and recruitment of stem cells. During the middle and late stages, inflammation inhibits and disrupts the differentiation of stem cells. In terms of the differentiation and expression fate of the latter, stem cells first differentiate into fibroblasts, then into chondrocytes, and finally into osteoblasts.^[^ ^55^, ^56^ ^]^ When the AIME is well regulated, inflammation rapidly resolves and the normal cartilage architecture is restored. When the AIME becomes imbalanced, rheumatoid arthritis, OA, hyperplastic osteophytes, or fibrotic scar tissues can develop, ultimately leading to the failure and death of the entire cartilaginous tissue.^[^ ^57^, ^58^, ^59^ ^]^ Therefore, maintaining homeostasis in the AIME as well as its proper intervention and control play crucial roles in cartilage repair and stem cell fate decisions. Temporal and spatial changes in the AIME are shown in **Figure** **2**.

### 3.1 Early Stage: Release of Danger Signals

The excessive and chronic release of damage‐associated molecular patterns (DAMPs) can lead to abnormal inflammation of the joints and destruction of the cartilage. In the AIME, alarm proteins (such as the high mobility group protein B1, heat‐shock proteins, IL‐33, and S100) are mainly derived from damaged or dead cells.^[^ ^60^, ^61^ ^]^ DAMPs primarily use the nuclear factor kappa B (NF‐κB) and mitogen‐activated protein kinase pathways to regulate immune and some nonimmune cells.^[^ ^60^, ^62^ ^]^ A decrease in S100 protein expression can significantly increase the activity of adipose stem cells.^[^ ^63^ ^]^ Conversely, an increase in S100 protein levels can induce stem cells to differentiate into osteoblasts and form ectopic osteophytes during the process of cartilage matrix remodeling.^[^ ^64^ ^]^ The excess accumulation of DAMPs leads to aging and a loss of stem cell activity, thereby leading to the adverse transformation of their cell fates, an imbalance of the AIME, and even the triggering of a systemic inflammatory reaction.^[^ ^62^, ^63^ ^]^ Additionally, during the early stage of cartilage injury, the dynamic balance of ROS in the AIME is disrupted. With the increase in the number of dead cells, ROS such as hydrogen oxide (H_2_O_2_), superoxide anion (O_2_^−^), and hydroxyl radical (OH)^−^ gradually accumulate in the AIME.^[^ ^65^ ^]^ Increased levels of reactive oxygen and nitrogen species (RONS) inhibit the proliferation and differentiation of MSCs by activating the NF‐κB pathway.^[^ ^66^, ^67^ ^]^ The accumulation of RONS can also inhibit the survival of MSCs and the secretion of type II collagen.^[^ ^68^ ^]^

### 3.2 Middle Stage: Protagonist Cells

Following cartilage injury, the innate immune response cells are activated first, while macrophages, monocytes, and multinuclear neutrophils invade local tissues and release soluble inflammatory factors.^[^ ^69^ ^]^ When the cartilage is injured, neutrophils, as an advance team, respond within 20 min and migrate within 2−6 h.^[^ ^70^, ^71^ ^]^ Under the stimulation of inflammation, neutrophils release neutrophil extracellular traps (NETs) within 60 min.^[^ ^72^ ^]^ Neutrophils respond to DAMPs, accumulate at the injured site, release matrix metalloproteinase (MMP)−9, and clear cell debris.^[^ ^71^, ^73^ ^]^ After the neutrophils mature, they are cleared by macrophages and home to the bone marrow. Mature neutrophils release liposomes to inhibit the entry of other neutrophils. By releasing TGF‐β, IL‐10, and other growth factors, they reprogram monocyte‐derived macrophages from the proinflammatory phenotype to the anti‐inflammatory phenotype.^[^ ^74^ ^]^ However, in cases of excess ROS accumulation or an unscheduled apoptosis of neutrophils, neutrophils release NETs to aggravate the tissue damage.^[^ ^72^, ^75^ ^]^ The timely clearance of neutrophils and the low‐level secretion of NETs are the first step toward the formation of a beneficial AIME.^[^ ^76^ ^]^ This is critical for the activation and recruitment of stem cells.

Mast cells, which are nonspecific immune cells, secrete MMPs, IL‐1, IL‐17, and other substances to recruit other immune cells when cartilage is injured, thereby promoting the degradation of dead chondrocytes and remodeling of the subchondral bone.^[^ ^77^ ^]^ Natural killer (NK) cells can stimulate the recruitment of MSCs by releasing chemokine (C‐X‐C motif) ligand 7 (CXCL7), CXCL2, CXCL3, chemokine (C‐C motif) ligand 5 (CCL5), and IL‐8, thereby helping to activate stem cells^[^ ^78^, ^79^ ^]^ and release interferon gamma (IFN‐γ) to resist further inflammation.^[^ ^80^ ^]^ Additionally, crosstalk between NK cells and MSCs affects the viability of MSCs to some extent.^[^ ^81^ ^]^

Macrophages play a key role in maintaining the homeostasis of the tissue microenvironment in the cartilage.^[^ ^82^, ^83^ ^]^ Macrophages in the articular cavity primarily come from the synovium, bone marrow, and IFP, including resident and recruited macrophages.^[^ ^84^, ^85^, ^86^ ^]^ The recruited major histocompatibility complex (MHC)‐II macrophages play a leading role in the regression of inflammation and reprogramming of the inflammatory phenotype.^[^ ^87^ ^]^ Within 72 h of cartilage injury, macrophages infiltrate the injured tissue and increase in numbers. Macrophages polarize to M1‐type macrophages (specific markers: CD80, CD86, CD40, and MHC‐II) and secrete chemokines and MMPs to clear dead cells. During the middle and late stages of inflammation, macrophages polarize to M2 macrophages (specific markers: ARG‐1, CD163, and CD206), secrete platelet‐derived growth factor and TGF‐β, and form an AIME that is conducive to the proliferation and differentiation of MSCs, while M2 macrophages promote the synthesis of ECM to facilitate wound contraction and healing.^[^ ^88^, ^89^, ^90^ ^]^ The timely transformation of the macrophage phenotype plays a decisive role in the formation of the AIME. M1 macrophage‐secreted tumor necrosis factor alpha (TNF‐α), IL‐1, and IL‐6 drive stem cells to differentiate into fibroblasts.^[^ ^90^ ^]^ However, studies have shown that TGF‐β, hypoxia‐inducible factor 1 alpha, IL‐4, and MMP12 are the main factors that drive macrophages to differentiate into (IL‐4) M subtypes and promote cell fibrosis.^[^ ^91^, ^92^, ^93^ ^]^ An increase in COL2A expression increases M2 macrophage differentiation, inhibiting apoptosis and the excessive hypertrophy of stem cells.^[^ ^94^ ^]^

In the AIME, T‐cells, B‐cells, and dendritic cells (DCs) mainly play the role of antigen presentation and chemokine secretion and indirectly regulate the joint microenvironment.^[^ ^87^ ^]^ Activated regulatory T (Treg) cells promote the expression of IL‐10 and TGF‐β and the secretion of indoleamine 2,3‐dioxygenase that inhibits the production of IL‐6, thereby maintaining the stability of the regenerative microenvironment and indirectly promoting tissue regeneration. In the AIME, CXCL10 binds to CXCR3 and cells that express CXCR3 (NK and activated T‐ and B‐cells) are attracted to the inflammatory site and indirectly affect the fate of stem cells. B‐cells secrete various proinflammatory factors (including IL‐1β, IL‐6, TNF‐α) and induce chondrocyte death and cartilage matrix rupture. Mature DCs activate Th1‐ and Th17‐cells, thereby inhibiting the chondrogenic development of MSCs, resulting in cartilage degradation. Immature DCs not only promote Treg cell proliferation but also trigger MSCs to differentiate into chondrocytes through the secretion of IL‐10.

### 3.3 Late Stage: Inflammatory Mediators

Within 24 h after injury, the content of inflammatory factors (IL‐1β, IL‐6, IL‐8, and TNF‐α) in the AIME increases significantly and peaks within 72 h. The content of these inflammatory factors can be higher than normal within 3 weeks of inflammation.^[^ ^95^, ^96^ ^]^ The degradation proteins of the cartilage matrix, such as the MMP family, reach their highest levels at 24 days after the peak expression of inflammatory factors. The recruitment factor of macrophages (CCL2) reaches its peak within 72 h after injury.^[^ ^97^ ^]^ In the late stage of AIME imbalance, the accumulation and persistence of inflammatory factors further stimulates inflammatory cells to cause a cytokine storm, which not only inhibits the activity of stem cells but also affects the original cartilage tissue and causes extensive inflammation in the joints.^[^ ^98^, ^99^ ^]^ Among these factors, IL‐1β, TNF‐α, IL‐6, and IL‐17 play a leading role in the AIME at the terminal stage. They can not only promote each other's release but also initiate the downstream inflammatory reaction.^[^ ^100^ ^]^ Both IL‐1β and TNF‐α inhibit the vitality of MSCs, reduce the production of glycosaminoglycan, increase the production of nitric oxide and MMPs, and further destroy the cartilage matrix.^[^ ^101^, ^102^ ^]^ In fact, IL‐1β and TNF‐α and the accumulation of IL‐6 and IL‐17 amplify osteoclast activity and accelerate the necrosis and fibrosis of damaged tissues.^[^ ^103^, ^104^, ^105^ ^]^ Furthermore, IL‐18,^[^ ^106^ ^]^ MMPs,^[^ ^107^ ^]^ and Yes‐associated protein^[^ ^108^ ^]^ can inhibit the differentiation of stem cells into cartilage.

## 4 Material Design Strategies

### 4.1 Silencing of Danger Signals

**Figure 3:**
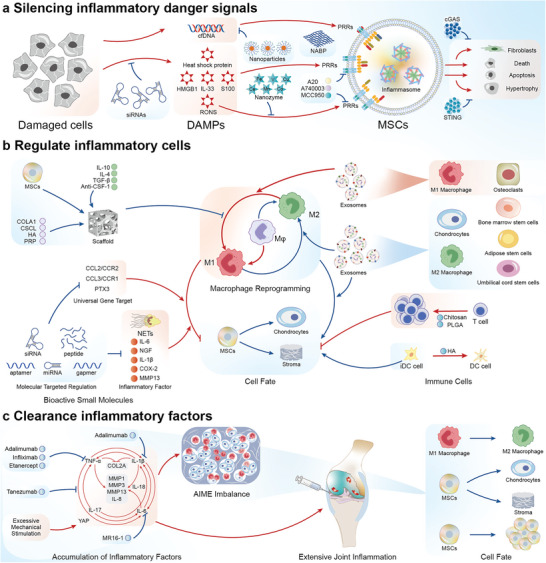
Designing materials that can guide stem cell fate. A) Silencing the inflammatory danger signal. Early danger signals are primarily produced by damaged cells, including DAMPs and cell‐free DNA (cfDNA). Cationic nanoparticles can clear the cfDNA. The expression of DAMPs can be silenced by siRNA. Nanozymes can clear the excess DAMPs. Nucleic acid‐binding polymers (NABPs), A20, A74003, and MCC950 inhibit pattern recognition receptors (PRRs), thereby reducing the production of inflammasomes in mesenchymal stem cells (MSCs). B) Regulation of inflammatory cells. The loading of stem cells and active molecules onto scaffolds can inhibit M2 to M1 macrophage conversion. Exosomes secreted by M1 macrophages and osteoclasts promote a shift in the AIME toward cartilage destruction. Exosomes secreted by chondrocytes and MSCs promote the AIME shift toward repair. Inflammatory factors in the AIME as well as universal gene targets can be targeted by bioactive small molecules. Chitosan and poly(D,L‐lactide‐*co*‐glycolide) (PLGA) can inhibit stem cell differentiation. Hyaluronic acid (HA) can promote immature DC (iDC) cell maturation and indirectly inhibit the differentiation of stem cells into cartilage. C) Clearance of inflammatory factors. Inflammatory factors not only enhance the secretion of one another but also promote the expression of MMPs and IL‐8 while inhibiting COL2A formation. Excess mechanical stress can increase the expression of YAP and promote the expression of inflammatory factors, which can be neutralized by specific antibodies.

In the event of early danger signals in the AIME, the excessive release of DAMPs can be inhibited by blocking their receptors or action processes (**Figure** **3**a). The risk of the formation of a detrimental AIME may be assessed by determining the S100 protein content in joints.^[^ ^54^ ^]^ DAMPs can activate multiple pattern recognition receptors (such as membrane‐bound Toll‐like receptors (TLRs) and C‐type lectin receptors, cytoplasmic NOD‐like receptors (NLRs), and retinoic acid‐inducible gene‐I‐like receptors) and some nonpattern recognition receptors (such as the receptor for advanced glycation end products, triggering receptors expressed on myeloid cells, and G‐protein‐coupled receptors).^[^ ^109^ ^]^ Therefore, these receptors can be targeted to inhibit inflammation progression and stem cell apoptosis. For example, A740003,^[^ ^110^ ^]^ MCC950,^[^ ^111^ ^]^ and A20^[^ ^112^ ^]^ can regulate the AIME in OA models in vivo by inhibiting the NLR family pyrin domain containing three inflammatory bodies. The improved nucleic acid‐binding polymers can neutralize the activation of TLRs caused by DAMPs.^[^ ^113^ ^]^ Moreover, some cationic nanoparticles can bind cell‐free DNA (cfDNA) to block TLR activation.^[^ ^114^, ^115^ ^]^ Liang et al. applied cationic nanoparticles to a rat model of rheumatoid arthritis and successfully relieved joint swelling and osteochondral injury.^[^ ^116^ ^]^ Additionally, small interfering RNA (siRNA) may also be designed to directly target the generation of DAMPs.^[^ ^117^ ^]^ Stimulator of interferon genes, cyclic GMP‐AMP synthase, and other small molecule inhibitors also eliminate the inflammatory reaction caused by DAMPs.^[^ ^118^, ^119^, ^120^ ^]^ Moreover, the generation of RONS can be eliminated through the use of active antioxidant materials. For example, nanoenzyme containing metal ions, such as Ce, Fe, Ce, Mn, V, Cu, and Co, can eliminate RONS through electron transfer with materials.^[^ ^121^, ^122^, ^123^, ^124^ ^]^ Li et al. successfully alleviated joint hypoxia in a rheumatoid arthritis model using a nanoenzyme doped with concave cubic rhodium (Rh/SPX‐HAS), thereby improving the AIME.^[^ ^121^ ^]^ Kim et al. cleared ROS in a rat model of rheumatoid arthritis using manganese ferrite/cerium co‐modified nanoparticles, thereby alleviating the progression of arthritis.^[^ ^124^ ^]^

### 4.2 Regulation of Protagonist Cells

#### 4.2.1 Biomolecule‐Mediated Regulation of Inflammatory Cells

In the AIME, macrophage reprogramming is a determinant of AIME condition (Figure 3b). The traditional method of regulating macrophages attaches inducible factors, such as IL‐10, IL‐4, and TGF‐β, as well as the neutralizing antibody for CSF‐1 to the biological scaffold to promote its positive effect on stem cells.^[^ ^125^, ^126^ ^]^ Bioactive factors, such as type I collagen, chondroitin sulfate, sulfated HA, and platelet‐rich plasma, can also be added to the material to indirectly induce the polarization of macrophages, promote the differentiation of stem cells, and protect the newly formed cartilage tissue from arthritis.^[^ ^127^ ^]^

#### 4.2.2 Exosome‐Mediated Regulation of AIME Macrophages

Exosomes derived from stem cells are AIME regulating substances produced via paracrine secretion and are an effective component of stem cell therapy. MSC‐derived exosomes can effectively reduce the production of inflammatory cytokines in chondrocytes, increase the expression of cartilage ECM components, and promote the regeneration of cartilage tissue.^[^ ^128^ ^]^ Recently, MSC‐derived exosomes have become excellent tools to regulate the AIME. Extracellular vehicles (EVs) derived from ADSCs, BMSCs, or embryonic stem cells can inhibit the production and infiltration of M1 macrophages, increase the expression of cartilage ECM components, and promote the differentiation of stem cells into cartilage.^[^ ^101^, ^128^, ^129^ ^]^ Woo et al. successfully inhibited M1 macrophage infiltration in rat and mouse OA models treated with MSC‐derived exosomes, thereby ameliorating OA symptoms.^[^ ^130^ ^]^ Jiang et al. used Wharton's jelly MSC‐derived exosomes to successfully promote cartilage regeneration and improve the joint cavity microenvironment in rabbit and rat cartilage defect models.^[^ ^131^ ^]^ Interestingly, the type of stem cells used can only affect the production of exosomes but not the efficiency.^[^ ^132^ ^]^ Some adult cells also secrete exosomes. The exocrine activity of osteoclasts promotes chondrocyte hypertrophy.^[^ ^133^ ^]^ Exosomes derived from M1 macrophages aggravate the inflammatory reaction in the AIME;^[^ ^134^ ^]^ however, those derived from chondrocytes and M2 macrophages exhibited the potential to promote cartilage repair. Sang et al. successfully promoted the transformation of macrophages in the AIME toward the M2 type by carrying exosomes derived from primary chondrocytes using thermosensitive hydrogels, thereby alleviating OA.^[^ ^135^ ^]^ Da‐Wa et al. used M2 macrophage‐derived exosomes to inhibit the PI3K/AKT/mTOR pathway in the AIME, thereby inhibiting the inflammatory response and minimizing knee OA damage in a rat model.^[^ ^136^ ^]^

#### 4.2.3 Repair Material‐Mediated Regulation of AIME Cells

Macrophage polarization refers to the process wherein a macrophage displays different phenotypes (M1 and M2) corresponding to a different AIME. The transformation of phenotypes between M1 and M2 macrophages is important in the regulation of stem cell fate. Based on macrophage polarization, we can regulate other immune cells to achieve the goal of macrophage reprogramming. Poly(D,L‐lactide‐*co*‐glycolide) or chitosan can accelerate the maturation of DC cells and increase the proliferation of T‐cells, release of inflammatory factors, and destruction of cartilage.^[^ ^89^ ^]^ HA and agarose can impede the maturation of DC cells, maintain the function and phenotype of DC cells at the level of immature DCs, and promote the differentiation of stem cells into chondrocytes.^[^ ^137^ ^]^

#### 4.2.4 Precise Regulation of Cell‐Specific Targeting

As highly programmable materials, small peptides and nucleic acids can precisely regulate cell behavior in the AIME through targeted delivery.^[^ ^138^ ^]^ For macrophages, many RNA array studies have revealed the targets of miRNA and mRNA capable of regulating the polarization of macrophages.^[^ ^139^, ^140^ ^]^ Additionally, internal universal gene targets associated with the AIME have been identified, namely, CCL2/CCR2,^[^ ^141^ ^]^ CCL3/CCR1,^[^ ^142^ ^]^ and PTX3.^[^ ^143^ ^]^ The selective targeting of these systems is a promising way to reprogram cell phenotypes in the AIME. The targeted delivery of siRNA and gapmer antisense oligodeoxynucleotides can significantly inhibit the expression of inflammatory factors in inflammatory cells. Bedingfield et al. could protect cartilage and improve the AIME in acute and advanced mouse OA models using siRNA‐loaded nanoparticle libraries.^[^ ^144^ ^]^ Chen et al. used interfering oligonucleotides and Au nanoparticles to construct spherical nucleic acids. They attached them to HA to inhibit OA‐induced cartilage cell degeneration in an in vivo OA model.^[^ ^145^ ^]^ They used aptamer RA10‐6 to directly bind the receptor of inflammatory factors, inhibit the expression of IL‐6 in the synovium of OA mice, and reduce inflammation.^[^ ^145^ ^]^ In the AIME, lncRNA and circRNA can be used as indicators to determine whether the AIME is conducive to stem cell growth.^[^ ^146^, ^147^ ^]^ Recently, Cruz et al. successfully achieved the targeted elimination of NETs through the use of α1‐antitrypsin‐derived peptides, thereby neutralizing the harmful pathological effects of neutrophils.^[^ ^148^ ^]^

### 4.3 Inhibition of Inflammatory Factors

During the late stage of inflammation, the use of a specific neutralizing antibody against inflammatory factors is an effective approach to alleviate inflammation (Figure 3c). Specific neutralizing antibodies, such as adalimumab,^[^ ^149^ ^]^ infliximab,^[^ ^150^ ^]^ anakinra,^[^ ^151^ ^]^ tanezumab,^[^ ^152^ ^]^ can be used against inflammatory factors to ameliorate the extensive inflammation of the AIME. Yi et al. successfully reduced cartilage degradation, synovial inflammation, osteophyte formation, and the pain caused by OA through administration of a neutralizing antibody against IL‐22.^[^ ^153^ ^]^ In clinical studies, nonsteroidal anti‐inflammatory drugs^[^ ^154^ ^]^ and hydroxychloroquine^[^ ^155^ ^]^ exerted therapeutic effects on the AIME. Additionally, increasing the levels of anti‐inflammatory mediators, such as IL‐4, IL‐10, and IL‐13, is an effective strategy.^[^ ^121^, ^156^ ^]^ In fact, Van Meegeren et al. successfully limited in vitro blood‐induced cartilage damage by combining IL‐4 with IL‐10.^[^ ^157^ ^]^

### 4.4 Drug Effectiveness Enhancement Strategies

**Figure 4:**
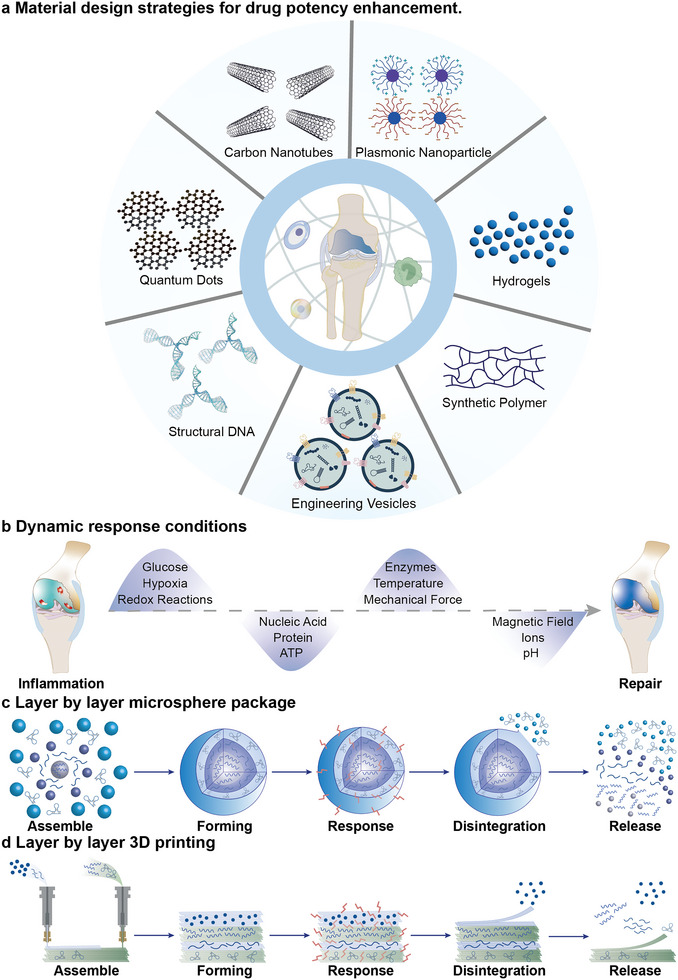
Material design strategies for enhancing drug potency. a) Carbon nanotubes (CNTs), QDs, structured DNA, engineered vesicles, synthetic macromolecules, hydrogels, and neutral particles are effective materials for delivering drugs to the cartilage. These materials can enhance the activity of functional molecules, improve the delivery concentration, and increase targeting of drug. b) Dynamic response microsphere. Microspheres are suitable for injection materials. After assembly and formation, they respond to different conditions in the environment. c) LbL 3D printing of the dynamic response, which is suitable for building a scaffold. 3D‐printed LbL materials have mechanical strength. d) Conditions for the dynamic response. The course of the AIME changes with dynamically changing materials, which enables self‐diagnosis and timely drug delivery. Responsive release can be achieved through the synthesis of multilayered nanospheres as well as through LbL 3D printing. These materials can respond to changes in glucose, nucleic acid, protein, ATP, and enzyme levels; hypoxia; redox reactions; and pH in the joint cavity. Temperature, mechanical force, magnetic field, and ions can also be applied externally to promote the active release of the drugs.

To enhance the function of small molecule drugs, the adoption of a design strategy to enhance efficacy is necessary (**Figure** **4**a). There are two enhancement strategies for small molecule drugs: 1) increasing the drug delivery concentration and 2) a drug targeting strategy (**Table** **2**).^[^ ^158^, ^159^, ^160^, ^161^, ^162^, ^163^, ^164^, ^165^, ^166^, ^167^, ^168^, ^169^, ^170^, ^171^, ^172^, ^173^, ^174^, ^175^, ^176^, ^177^, ^178^, ^179^, ^180^, ^181^, ^182^, ^183^, ^184^, ^185^, ^186^, ^187^, ^188^, ^189^, ^190^, ^191^, ^192^, ^193^, ^194^, ^195^, ^196^, ^197^, ^198^, ^199^, ^200^, ^201^, ^202^, ^203^, ^204^, ^205^, ^206^, ^207^, ^208^, ^209^, ^210^, ^211^ ^]^ Andersen et al. attempted to target MTX and siRNA in neutrophils and monocytes using CNTs.^[^ ^160^ ^]^ Jeyadevi et al. used thioglycolic acid‐CdTe quantum dots (QDs) as a carrier to deliver quercetin for the treatment of rat OA.^[^ ^167^ ^]^ Although these two materials are characterized by an excellent delivery performance, their hepatotoxicity remains a problem. As a highly programmable biocompatible material, structural DNA can achieve good targeting through the modification of aptamers. Ma et al. achieved a targeted antioxidant effect with DNA origami drugs.^[^ ^170^ ^]^ Engineering vesicles have the advantages of immune escape and low immunogenicity. Moreover, they can carry a wide range of molecules and effectively increase the delivered concentration. Li et al. used EVs to carry curcumin and to alleviate the progress of OA.^[^ ^212^ ^]^ Hydrogels, as a widespread and low toxicity molecular delivery material, have many advantages: they can be widely sourced, exhibit good biocompatibility, and are easy to synthesize. Yu et al. used hydrogel microspheres to repair cartilage in an OA model. Synthetic polymers can significantly prolong the retention time of drugs and determine the action time of drugs based on charge.^[^ ^188^ ^]^ Shen et al. used a synthetic polymer to wrap dexamethasone and achieve targeted and biological imaging of osteoarthritis.^[^ ^208^ ^]^ Plasma nanoparticles, such as gold nanoparticles (AuNPs), have obvious advantages for the delivery of negatively charged materials. Li et al. used AuNPs to develop an antioxidative and anti‐inflammatory treatment and reduced the toxicity of the AuNPs through surface modification.^[^ ^209^ ^]^

**Table 2:** Material design strategies for drug efficiency enhancement

	Examples	Drug delivery category	Size	Features	References
Carbon nanotubes	Single‐wall carbon nanotubes (SWCNTs) Multiwall carbon nanotubes (MWCNTs)	DMARDs^a)^ siRNA^a)^ Corticosteroids	1 nm	High drug loading efficiency Smaller diameter than the cartilage meshwork size Higher uptake efficiency Hepatotoxicity	[158, 159, 160, 161, 162, 163]
Quantum dots	CdSe/CdS/ZnS QDs Graphene QDs (GQDs)	Natural polymer	1–15 nm	Traceable drug delivery Photochemical stability Hepatotoxicity	[164, 165, 166, 167]
Structural DNA	DNA origami structure	siRNA^a)^ Therapeutic proteins	1–100 nm	Excellent biocompatibility Programmability Enhance drug targeting and cell uptake efficiency Scavenge proinflammatory factor	[168, 169, 170]
Engineering vesicles	Microvesicle Exosomes Liposomes Cell membrane	miRNA^a)^ siRNAa) DMARDs^a)^ Natural polymer Corticosteroids	30–200 nm	Low toxicity Low immunogenicity High engineerability Escape immune recognition	[171, 172, 173, 174, 175, 176, 177, 178, 179, 180, 181, 182]
Hydrogels	GelMA hydrogels Hyaluronic acid hydrogels Supramolecular hydrogels Chitosan hydrogels Hydrogel microspheres	DMARDs^a)^ Natural polymer Exosome Corticosteroids plasmid DNA^a)^	–	Exquisitely tunable physical properties Injectability Drug sustained release Drug protection Low toxicity Low immunogenicity Excellent biocompatibility	[183, 184, 185, 186, 187, 188, 189, 190, 191, 192]
Synthetic polymer	PLGA^a)^ PEG^a)^ Dendrimers PCADK^a)^ PAMAM^a)^	siRNA^a)^ Therapeutic proteins NSAIDs DMARDs^a)^ Natural polymer Corticosteroids	100–300 nm	Size‐related penetration Prolonged drug retention time Charge‐dependent drug retention	[193, 194, 195, 196, 197, 198, 199, 200, 201, 202, 203, 204, 205, 206, 207, 208]
Plasmonic nanoparticle	AuNPs^a)^	siRNA Corticosteroids	1.5 nm to 1 µm	Hepatotoxicity Clear efficiency dependency Surface modification dependency	[209, 210, 211, 212, 213, 214, 215, 216, 217, 218, 219, 220, 221]

^a)^ AgNPs, silver nanoparticles; AuNPs, gold nanoparticles; DMARDs, disease‐modifying antirheumatic drugs; HA, hyaluronic acid; NSAIDs, nonsteroidal anti‐inflammatory drugs; PAMAM, poly(amidoamine); PCADK, poly(cyclohexane‐1,4‐diyl acetone dimethylene ketal); PEG, poly(ethylene glycol); PLGA, poly(D,L‐lactide‐*co*‐glycolide); siRNA, small interfering RNA; QDs, quantum dots; miRNA, micro RNA; DNA, deoxyribonucleic acid; RNA, ribonucleic acid.

### 4.5 Spatial and Temporal Regulation Strategy

With the maturity of AIME‐targeting technology, methods for regulating the AIME have improved. A dynamic regulation strategy of the inflammatory response based on nanomaterials can regulate the balance of the AIME.^[^ ^213^ ^]^ Dynamic response materials can determine the response time point according to pH, redox reaction, enzyme, glucose, ions, adenosine triphosphate (ATP), anoxic environment, temperature, mechanical stress, and nucleic acids, which are parameters beyond the reach of conventional drugs (Figure 4b).^[^ ^214^ ^]^ Such materials can deliver and effectively release most molecules, including proteins, nucleic acids, and therapeutic drugs. To adapt to the temporal and spatial changes occurring during inflammation, the layer‐by‐layer (LbL) technology is gradually becoming the main choice for delivering precision medicine (Figure 4c,d).^[^ ^215^ ^]^ Electrohydrodynamic jet 3D printing,^[^ ^216^ ^]^ low‐temperature deposition 3D printing,^[^ ^217^ ^]^ and microfluidic technology^[^ ^218^ ^]^ are effective means for achieving this goal. The biomaterials designed using LbL technology can adapt to the temporal and spatial changes of the AIME as well as to the different anatomical structures of the defect, thereby enabling the delivery of various bioactive molecules.

## 5 Perspectives

**Figure 5:**
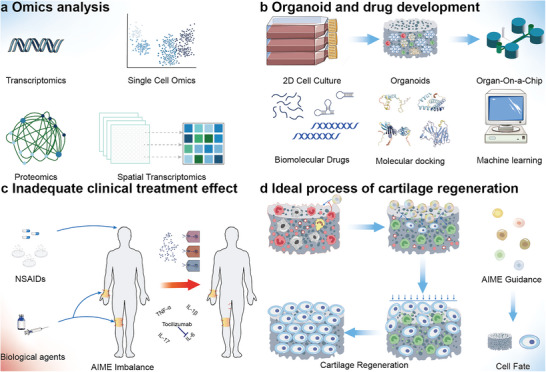
Further understanding of the AIME and drug development. a) Multiomics research. Transcriptomics, proteomics, single‐cell omics, and spatial transcriptomics can illustrate the evolution of the AIME through multiple dimensions. b) Organoid technique studies. Culture of joint cavity cells in a 2D environment. The cultured cells were implanted into a 3D ECM. Microfluidic chips reconstruct the pressure environment of the joint cavity cells. The organoid technique enables the development and validation of novel small molecule drugs through molecular docking and machine learning. c) Unsatisfactory results of clinical treatment. NSAIDs and drugs antagonizing a single molecule cannot reverse AIME deregulation. Insufficient intracellular drug concentrations and the inhibition of a single molecule may be responsible for this failure. d) Ideal process for achieving cartilage regeneration. The early phase includes the release of DAMPs, recruitment of macrophages to clear the necrotic tissue, and inhibition of stem cell adhesion. The middle phase is dominated by M2 macrophages, which guide the recruitment and differentiation of stem cells. The late phase involves the clearing of inflammatory factors and tissue remodeling (assisted by mechanical forces). The different stem cells, as guided by an ideal AIME, realize the greatest potential of their fate trends, differentiate into chondrocytes and matrix, and repair the cartilage.

Different AIMEs can play a decisive role in cell fate. Herein, we have discussed the influence of various factors on stem cell fate. An effective method to simulate this complex microenvironment is by constructing cartilage organoids and combining transcriptomics, proteomics, spatial transcriptomics, and machine learning methods to analyze and model the AIME (**Figure** **5**a).^[^ ^219^ ^]^ Moreover, organ‐on‐a‐chip engineering technology based on microfluidics and 3D printing will enable further exploration of the influence of the AIME on specific cells (Figure 5b). More importantly, organoid technology can simulate and evaluate the efficacy of new drugs, thereby providing more data for the clinical transformation of a new generation of cartilage‐targeting drugs.^[^ ^220^ ^]^

The use of a previous generation of biomaterials has generated significant knowledge. For example, in animal models, key factors of the AIME and novel therapeutic strategies have been verified. However, in clinical studies, the strategy of a single molecule‐targeted antagonism has not achieved satisfactory results (Figure 5c).^[^ ^100^, ^221^ ^]^ One reason may be that the drug delivery strategy leads to the inability of inflammatory factors to target the desired site, thereby resulting in ineffective concentrations of inflammatory factors. Another reason may be that the inhibition of a single molecule is not sufficient to reverse the progress of the AIME imbalance during the late stage of inflammation. Delivering effective biomolecules with different enhancement strategies is an effective method for increasing drug concentration and targeting efficiency.^[^ ^222^ ^]^ However, evaluating the safety of small molecular particles and nanoparticles as well as the uncertainty of their therapeutic effects are factors that hinder their clinical transformation. Recently, bioactive molecules based on structured DNA and active peptides have exhibited great potential for use in cartilage repair. The deconstruction of small molecule peptides and development of methods for simulating nucleic acid molecular dynamics have provided a large research library for constructing new protein and nucleic acid molecules. We believe that the development of novel small molecule drugs will be significantly accelerated in the future.

## 6 Conclusions

The potential for cartilage regeneration is closely associated with the AIME. An ideal AIME following injury should progress through three stages (Figure 5d). The early AIME should release danger signals that recruit M1 macrophages to clear the necrotic tissue and inhibit the attachment of stem cells. In the second phase, M2 macrophages should dominate the AIME to recruit and induce the differentiation of stem cells, which promote the initial formation of cartilage tissue. Finally, in the late AIME stage, clearing of inflammatory cells and factors, remodeling of the cartilage morphology under mechanical forces, and the re‐establishment of the AIME should occur. With regard to stem cells with different fates, the AIME should be regulated to guide the stem cells such that they can exert their maximum potential and differentiate into cartilage. To cope with the dynamically changing microenvironment of the AIME, designing responsive and multilayer materials is important for the restoration of a normal AIME and to guide stem cell fate. As an active participant in the AIME change process, the patterning of dynamic materials is an important trend that should be adopted in future design attempts to develop stem cell fate‐guiding materials. We believe that guiding the fate of stem cells by regulating the AIME will serve as a promising strategy to create the next generation of cartilage repair materials.

## Conflict of Interest

The authors declare no conflict of interest.
